# 449. Performance of the Brighton Case Definition for Multisystem Inflammatory Syndrome in Children (MIS-C) Among a Large Single Center Cohort

**DOI:** 10.1093/ofid/ofab466.648

**Published:** 2021-12-04

**Authors:** Jessica Nguyen, Isabella Osuna, Eyal Muscal, Kristen Sexson, Marietta DeGuzman, Flor M Munoz, Tiphanie Vogel

**Affiliations:** 1 Baylor COM, Houston, Texas; 2 Rice University, Houston, Texas; 3 Baylor College of Medicine, Houston, Texas

## Abstract

**Background:**

Multisystem Inflammatory Syndrome in Children (MIS-C) is a rare, life-threatening, hyperinflammatory condition presumed to follow SARS-CoV-2 infection. Whether MIS-C can also follow SARS-CoV-2 vaccination is not clear, making MIS-C an adverse event of special interest following immunization. Monitoring for post-vaccine MIS-C is complicated by the clinical overlap of MIS-C with numerous other inflammatory conditions including Kawasaki Disease, toxic shock syndrome, and viral myocarditis. A case definition for MIS-C was recently created with the Brighton Collaboration (BC). We aimed to determine the performance of the BC MIS-C case definition among a large, single-center MIS-C cohort.

**Methods:**

Retrospective review was performed for the first 100 MIS-C cases at our institution (May 2020-February 2021). All cases met the Centers for Disease Control and Prevention (CDC) case definition. Data on age, presentation, laboratory results and cardiac studies were collected and used to determine cases that fulfilled the BC case definition for MIS-C (see figure).

Case Definition: Definite Case

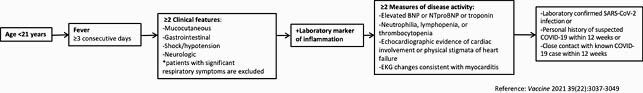

**Results:**

Of 100 children (age < 21 years) diagnosed with MIS-C using the CDC case definition, 93 patients also fulfilled the BC definition. All 100 patients had elevated laboratory markers of inflammation and positive SARS-CoV-2 antibodies. However, 1 patient was excluded for significant respiratory symptoms (pulmonary hemorrhage), 5 were excluded due to only 1 clinical feature, and an additional patient was excluded for having none of the measures of disease activity. Among the 93 patients fulfilling the revised case definition, 88 (95%) met criteria for a definite case. Five of the 93 patients (5%) were considered probable cases, 1 reported only 1 day of fever and 4 had only 1 measure of disease activity.

**Conclusion:**

The original case definitions for MIS-C were created rapidly following the first emerging reports of this hyperinflammatory state. Knowledge of the varied clinical presentations of this disorder has grown substantially. Modification of the case definition to include features truly representative of MIS-C will allow for more precise diagnosis in the face of conditions which mimic MIS-C, and for accurate and reliable monitoring for adverse events following immunization.

**Disclosures:**

**Flor M. Munoz, MD**, **Biocryst** (Scientific Research Study Investigator)**Gilead** (Scientific Research Study Investigator)**Meissa** (Other Financial or Material Support, DSMB)**Moderna** (Scientific Research Study Investigator, Other Financial or Material Support, DSMB)**Pfizer** (Scientific Research Study Investigator, Other Financial or Material Support, DSMB)**Virometix** (Other Financial or Material Support, DSMB)

